# Combined repetitive transcranial magnetic stimulation and medication treatment for depression is associated with serum amyloid a level: Evidence from naturalistic clinical practice

**DOI:** 10.3389/fnins.2022.1002816

**Published:** 2022-09-14

**Authors:** You Xu, Li Han, Youdan Wei, Hongjing Mao, Zhenghe Yu

**Affiliations:** Department of Sleep Medicine, Affiliated Mental Health Center and Hangzhou Seventh People’s Hospital, Zhejiang University School of Medicine, Hangzhou, China

**Keywords:** rTMS, depression, HAMD, SAA, testosterone

## Abstract

**Objective:**

Repetitive transcranial magnetic stimulation (rTMS) has a positive effect on patients with depressive disorder, while the underpinning molecular mechanism is unknown. Here, we aimed to investigate the effect of rTMS on serum levels of serum amyloid A (SAA) and testosterone in a real-world setting.

**Materials and methods:**

In total, ninety-seven patients with depressive disorder were treated with medicine and rTMS (the rTMS group) while 122 patients were treated using the medicine only (the control group). Plasma levels of SAA (*n* = 52) and testosterone (*n* = 37) were measured before and after 2 weeks of treatment, and the treatment effect was evaluated by Hamilton Rating Scale for Depression (HAMD).

**Results:**

The treatment effect revealed by the percentage of decrease in HAMD in the second week was significantly greater in the rTMS group compared with the control group. No significant difference was found in SAA or testosterone levels between the two groups. However, the percentage of changes in SAA (*r* = −0.492, *p* = 0.017) in the second week was significantly correlated with the percentage of decrease in HAMD score in the rTMS group, but not in the control group.

**Conclusion:**

Patients with depression benefit more from combined rTMS and medication treatment in this naturalistic study. Changes in SAA level, but not testosterone level, were related to depressive remission after 2 weeks’ combined treatment.

## Introduction

Depression is a common psychiatric disorder with high lifetime prevalence, affecting up to 15% of the world’s population (Moussavi et al., 2007). Depressive disorders, such as major depressive disorder (MDD) and dysthymic disorder, are psychiatric illnesses with devastating personal and social consequences owing to a persistent depressed mood, negative thoughts, and fatigue. The WHO (World Health Organization, 2017) has declared depression to be the leading cause of disability worldwide. Current pharmacologic treatment options show limited effectiveness in countering the disease (Turner et al., 2008; Cipriani et al., 2018), and approximately 30% of patients do not experience sustained symptomatic remission despite multiple treatment attempts (Rush et al., 2006).

Repetitive transcranial magnetic stimulation (rTMS) is a non-invasive neuromodulation technique with broad clinical applications. A significant positive effect of rTMS on adult MDD patients has been demonstrated in several studies (McNamara et al., 2001; Burt et al., 2002; Herrmann and Ebmeier, 2006; O’Reardon et al., 2007). In current clinical practice, the left unilateral dorsolateral prefrontal cortex (DLPFC) 10 Hz stimulation protocol has been approved by the food and drug administration (FDA) for treatment-resistant depression patients. However, a meta-analysis suggested that the efficacy was not robust across studies or participants (Hyde et al., 2022). Full elucidation of the antidepressant mechanism of rTMS may help to explain the heterogeneity, and increase the chance of discovering new therapeutic strategies. A recent review (Luan et al., 2020) summarized the anti-depressant mechanism of rTMS in preclinical studies, namely, anti-inflammatory effects, anti-oxidative stress effects, enhancement of synaptic, and neurogenesis, the increased content of monoamine neurotransmitters, and the reduced activity of the hypothalamic-pituitary-adrenocortical axis. Another review has shown that the rTMS may exert a neuroprotective effect by acting on neuroinflammation in animal models of depression (Yulug et al., 2016). When unclear factor-E2-related factor 2 (Nrt2), which has an anti-inflammatory effect, was silenced, the antidepressant effect produced by the rTMS was abolished (Tian et al., 2020). The mechanism that rTMS effectively reverse despair-like behavior in rats could be related to regulating metabotropic glutamate receptors 5 (mGluR5)/N-Methyl-D-Aspartic acid receptor type 2B (NMDAR2B)-related inflammatory signaling pathways in the anterior agranular insular (Hu et al., 2022).

Several inflammatory markers, namely interleukin-1β (IL-1β), IL-6, and C-reactive protein (CRP), are associated with depression (Howren et al., 2009; Zunszain et al., 2013). Serum amyloid A (SAA), like CRP, is an acute-phase plasma protein, synthesized predominantly by the liver and induced by IL-1β and IL-6 (Moshage et al., 1988; Smith and McDonald, 1992; Eklund et al., 2012). Elevated levels of SAA have been detected in the plasma of patients with clinical depression compared with healthy controls (Wang et al., 2016). Another population-based cohort study has found that patients with depressive disorders had higher plasma SAA concentrations relative to individuals without such disorders (van Dooren et al., 2016). Plasma SAA was closely associated with depression severity across diagnostic boundaries in a naturalistic outpatient psychiatric sample (Bryleva et al., 2017). Serum levels of inflammatory cytokines such as IL-1β, IL-6, and tumor necrosis factor (TNF)-α were found to decrease after rTMS intervention (Zhao et al., 2019; Perrin and Pariante, 2020; Liu et al., 2022), which suggested that the antidepressant effect of rTMS may be related to changes in inflammatory (Wang et al., 2022). Besides, partial improvement of cognitive dysfunction by rTMS might be attributable to the reduction of peripheral IL-1β levels (Tateishi et al., 2020). Thus, SAA may be a part of the molecular mechanism of rTMS efficacy.

On the other hand, the association between testosterone and depression has been extensively debated because testosterone is a neuroactive steroid hormone influencing mood (Amiaz and Seidman, 2008). A population-based, longitudinal study showed inverse associations between androgens and depressive symptoms, although the associations were not independent of relevant confounders (Kische et al., 2017). In another longitudinal study on children, the rTMS was effective in remediating testosterone to levels seen in age-matched controls (Bolotova et al., 2017). Besides, gonadal steroids are involved in regulating cortical excitability induced by rTMS (Bonifazi et al., 2004). Exogenous application of testosterone can also modify connectivity between the DLPFC and the amygdala, which is related to emotion regulation (Votinov et al., 2020). Based on these, we speculated that testosterone was also a potential molecular mechanism or an indicator of rTMS efficacy.

In the present study, we aimed to verify the effectiveness of combined rTMS and medication depression therapy in real-world clinical settings, and investigate the effect of rTMS on serum levels of SAA and testosterone in depression patients.

## Materials and methods

### Participants

**FIGURE 1:**
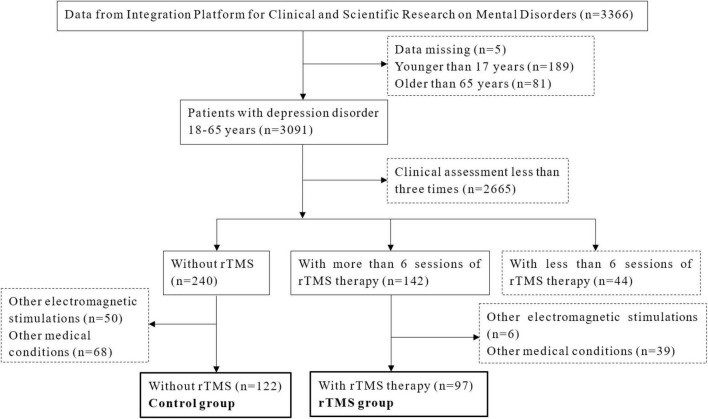
Flow diagram of the study.

This study included inpatients from the Affiliated Mental Health Center and the Hangzhou Seventh People’s Hospital, Zhejiang University School of Medicine. All the data were acquired from the Integration Platform for Clinical and Scientific Research on Mental Disorders. In total, 3,091 patients aged between 18 and 65 years, were diagnosed with depressive disorder by two treating psychiatrists according to the international classification of diseases, tenth (ICD-10) revision. Those who completed more than six sessions of rTMS (except for the control group) and finished clinical assessment of the Hamilton Rating Scale for Depression (HAMD) three times would be included in this study. Exclusion criteria were: received other electromagnetic stimulations such as electroconvulsive therapy; depression caused by other severe psychiatric disorders; history of severe somatic diseases and organic diseases of the brain; and having medication other than antidepressants, benzodiazepines/non-benzodiazepines, or low-dose of olanzapine/quetiapine. The flow chart of the study design is shown in Figure 1. A total of 219 patients were enrolled in this study, with 122 in the control group and 97 in the rTMS group. There were 52 patients (23 from the rTMS group and 29 from the control group) who measured SAA and 37 patients (20 from the rTMS group with 16 women, and 17 from the control group with 14 women) who measured testosterone at baseline and second week. The study protocol was approved by the ethics committee of the local hospital. Informed consent was obtained and the study abided by the Declaration of Helsinki principles.

### Repetitive transcranial magnetic stimulation treatment

All the rTMS treatment was administered by trained medical doctors. All the patients were seated in a comfortable chair while TMS stimuli were delivered to the left prefrontal cortex (using the 5-cm rule) with a figure-of-eight coil and an OSF-6 magnetic stimulator (Wuhan Aosaifu Medical Technology Co., Ltd., China). The patients received 5 sessions of rTMS treatment per week and the stimulation frequency was 10 Hz with power (intensity) level of 90% of motor threshold (MT). Each session contained 60 rTMS trains with 40 pulses per train and the intertrain interval was 15 s.

### Clinical assessment

The primary outcome of the study was the percentage of decrease in scores on the 24-item version of the HAMD. The outcome measure of HAMD was assessed at baseline (before rTMS treatment), first week (5 sessions), and second week (10 sessions). Response to treatment was defined as an over 50% decrease in HAMD. Remission was defined as a HAMD score of less than 8 in the second week.

### Blood sampling procedures and analyses

The blood sample was collected between 7:00 and 9:00 a.m. in a fasting state. Analyses of SAA and testosterone were performed on fresh biospecimens on the day of sample collecting. The SAA was analyzed using a particle-enhanced turbidimetric immunoassay (PETIA) and testosterone was analyzed using chemiluminescence analysis (CLIA).

### Data analysis

Data were analyzed using standard descriptive statistics in PASW Statistics 18.0 (SPSS Inc., Chicago, IL, United States) statistical software. The control group (*n* = 122) included patients with medical treatment while the rTMS group (*n* = 97) included those with joint medicine and rTMS treatments. Chi-square tests were used to investigate differences in men/women between groups. Repeated two-way ANOVA (group * time) was conducted to investigate the HAMD score/percentage of decrease in HAMD/SAA level/testosterone level difference between groups across 2 weeks of measures. Student’s *t*-test were used to investigate differences between groups in the percentage of changes in HAMD score, SAA, and testosterone level. Pearson correlation was used to investigate the relationship between the percentage of changes in SAA, testosterone levels, and HAMD score.

## Results

### Demographics, Hamilton Rating Scale for Depression score, serum amyloid A, and testosterone statistics

As shown in Table 1, the distributions of sex and age did not differ between the control and the rTMS groups. The percentage of patients who used benzodiazepine/olanzapine/quetiapine did not differ between the two groups either. The HAMD score at baseline and the first week did not differ between the two groups. However, the rTMS group scored lower than the control group on HAMD in the second week.

**TABLE 1:** Demographic, Hamilton Rating Scale for Depression (HAMD) score, serum amyloid A (SAA), and testosterone in the control and the repetitive transcranial magnetic stimulation (rTMS) groups.

	Control group (*n* = 122)	rTMS group (*n* = 97)	t/χ^2^	*P*
Age (years) [mean (SD)]	48.3 (11.5)	45.3 (11.8)	1.879	0.062
Age range (years)	18–65	22–65		
Female [*n* (%)]	91 (74.6)	76 (78.4)	0.516	0.528
Benzodiazepine [*n* (%)]	120 (98.4)	96 (99.0)	0.148	0.586
Olanzapine/Quetiapine [*n* (%)]	90 (73.8)	62 (63.9)	2.471	0.077
**Baseline assessments**				
HAMD [Mean (SD)]	23.5 (6.4)	23.5 (5.6)	0.024	0.981
SAA **(*n* = 52)**	7.5 (1.2)	9.3 (9.7)	–0.771	0.444
Testosterone **(*n* = 37)**	3.0 (5.6)	2.7 (4.2)	0.156	0.877
**First-week assessment**				
HAMD [Mean (SD)]	14.5 (4.9)	13.5 (4.4)	1.561	0.120
Decrease in HAMD [Mean (SD)]	9.1 (4.6)	10.1 (4.6)	–1.570	0.118
Percentage of decrease in HAMD [%]	37.6	42.5	–2.088	0.038
**Second-week assessment**				
HAMD [Mean (SD)]	7.5 (4.0)	6.4 (3.3)	2.098	**0.037**
Decrease in HAMD [Mean (SD)]	16.1 (5.2)	17.1 (5.7)	–1.404	0.162
Percentage of decrease in HAMD [%]	68.2	72.2	–2.112	**0.036**
Remission rate (%)	59.0	66.0	1.113	0.180
Response rate (%)	87.7	87.6	<0.001	0.573
SAA **(*n* = 52)**	8.7 (11.5)	7.0 (5.0)	0.673	0.504
Testosterone **(*n* = 37)**	2.9 (5.3)	3.3 (5.8)	–0.184	0.855

Bold values indicates a significant difference at the *p* = 0.05 level.

### Effectiveness of repetitive transcranial magnetic stimulation

**FIGURE 2:**
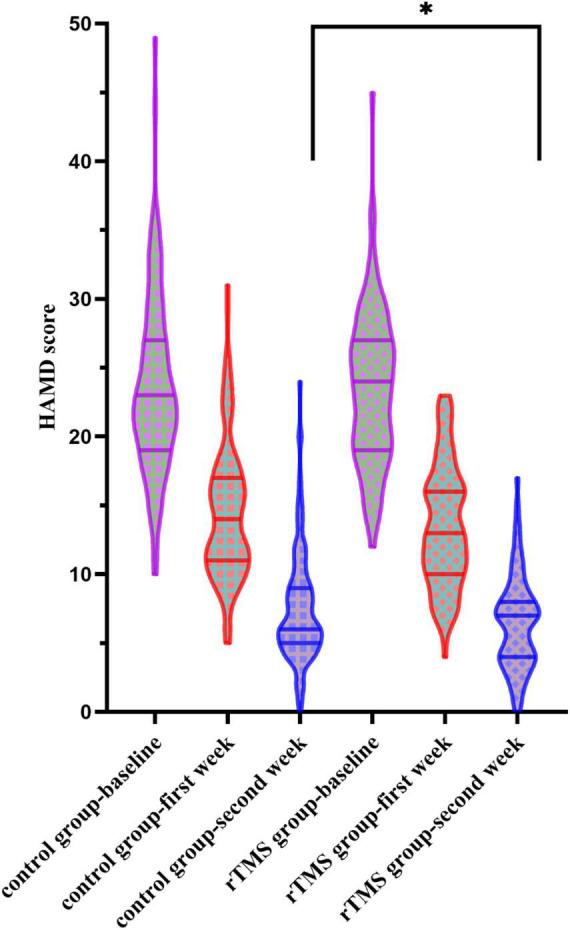
Hamilton Rating Scale for Depression (HAMD) scores at the baseline, the first week, and the second week in the control and the repetitive transcranial magnetic stimulation (rTMS) group. *Indicates a significant difference at the *p* = 0.05 level.

When conducting the two-way ANOVA statistic with group factors (rTMS/Control) and time factors (baseline, first week and second week) within HAMD score, there was a significant effect in time [*F*_(2,434)_ = 1431.734, *p* < 0.001] but not in the group [*F*_(1,217)_ = 1.506, *p* = 0.221] and interaction [*F*_(2,434)_ = 1.753, *p* = 0.175] (Figure 2). When conducting the two-way ANOVA statistic with group factors (rTMS/Control) and time factors (first week and second week) within percentage of decrease in HAMD, there were significant effects both in group [*F*_(1,217)_ = 5.799, *p* = 0.017] and time [*F*_(1,217)_ = 809.113, *p* < 0.001], but not in interaction [*F*_(1,217)_ = 0.143, *p* = 0.706], suggesting the effectiveness of rTMS treatment along the time. Further *t*-test showed that the percentage of decrease in HAMD in the rTMS group was significantly greater than in the control group in the first [*t*_(217)_ = −2.088, *p* = 0.038] and second week [*t*_(217)_ = −2.112, *p* = 0.036]. However, the response rate or remission rate of the rTMS group did not differ from the control group in the second week.

### No differences in serum amyloid A/testosterone levels between the control and the repetitive transcranial magnetic stimulation groups

**FIGURE 3:**
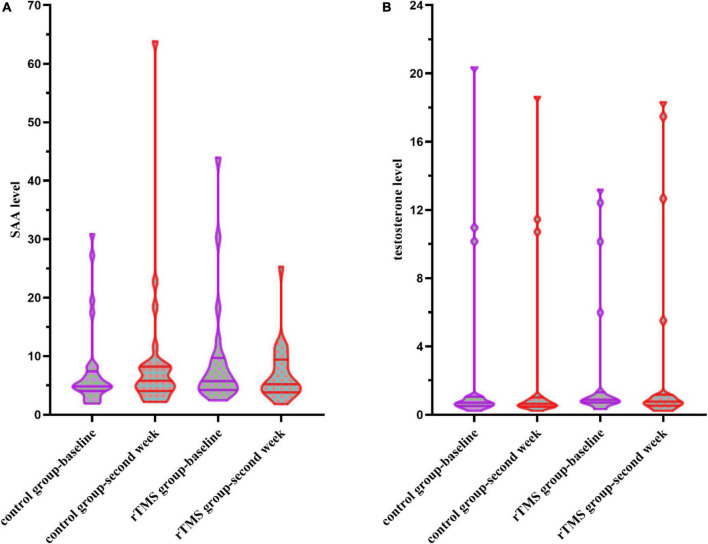
**(A)** Serum amyloid A (SAA) levels at the baseline and the second week in the control group and the rTMS group. **(B)** Testosterone levels at the baseline and the second week in the control group and the rTMS group.

When conducting the two-way ANOVA statistic with group factors (rTMS/Control) and time factors (baseline and second week) within SAA level of 52 patients, there was no significant group effect [*F*_(1,51)_ = 0.696, *p* = 0.408], time effect [*F*_(1,51)_ = 0.871, *p* = 0.355], or interaction effect [*F*_(1,51)_ = 0.242, *p* = 0.625] (Figure 3A). The percentage of decrease in SAA level [*t*_(50)_ = 1.550, *p* = 0.128] did not differ between control and the rTMS group.

When conducting the two-way ANOVA statistic with group factors (rTMS/Control) and time factors (baseline and second week) within testosterone level of 37 patients, there was no significant group effect [*F*_(1,35)_ = 0.001, *p* = 0.979], time effect [*F*_(1,35)_ = 0.791, *p* = 0.380], or interaction effect [*F*_(1,35)_ = 1.313, *p* = 0.260] (Figure 3B). The percentage of changes in testosterone level [*t*_(35)_ = 0.671, *p* = 0.507] did not differ between control and the rTMS group.

### Relationships between serum amyloid A/testosterone level changes and Hamilton Rating Scale for Depression decrease

**FIGURE 4:**
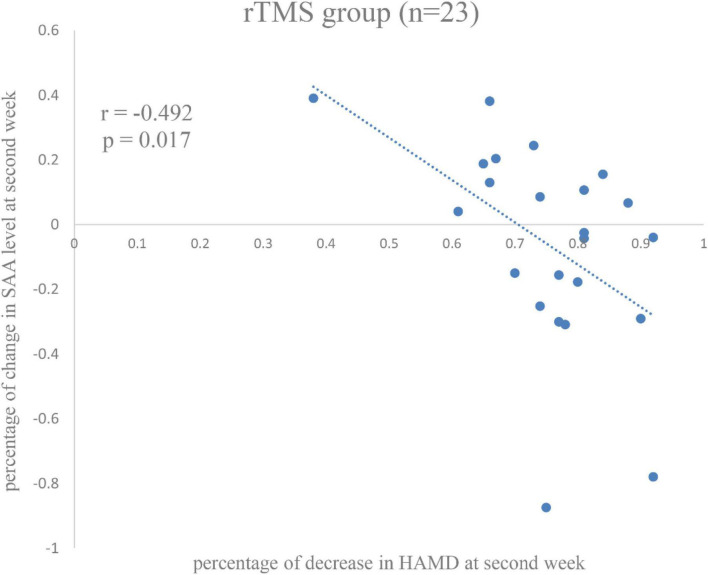
Correlation between percentage of changes in serum amyloid A (SAA) level and percentage of decrease in Hamilton Rating Scale for Depression (HAMD) score in the repetitive transcranial magnetic stimulation (rTMS) group at the second week.

The significant relationship was found between percentage of decrease in SAA level and the percentage of decrease in HAMD score in the rTMS group at second week (*r* = −0.492, *p* = 0.017) (Figure 4), rather than the control group (*r* = 0.105, *p* = 0.579), or among all patients (*r* = −0.025, *p* = 0.858).

Notably, no relationship was found between the percentage of change in testosterone level at second week and the percentage of decrease in HAMD score, neither in all patients (*r* = −0.071, *p* = 0.675) nor in separate groups (the rTMS group: *r* = −0.214, *p* = 0.366; the control group: *r* = 0.267, *p* = 0.299).

## Discussion

In this study, we found a greater percentage of decrease in HAMD score in the second week when combined with rTMS treatment than medical treatment only in depression patients, and the percentage of decrease in HAMD score was associated with the percentage of changes in SAA level in the second week.

The rTMS could accelerate the onset time of beneficial treatment effects and improve clinical symptoms of depression (Dai et al., 2022). In a study of depression patients who were administrated with drugs combined with rTMS treatment, the active rTMS group demonstrated a more significant score reduction compared to the sham rTMS group in the second week (Dai et al., 2022). Here, rTMS also showed early effectiveness in the second week. Research has indicated that benzodiazepines (BZD) may impede the response to rTMS (Deppe et al., 2021). Although most patients in this study took BZD during rTMS treatment due to insomnia, rTMS still showed its effectiveness within 2 weeks.

Although no significant result was found on the SAA level during the 2 weeks of combined rTMS treatment, the percentage of decrease in SAA level was related to the percentage of decrease in HAMD score. Changes in inflammatory mediators such as SAA were related to insomnia (Xia et al., 2021), which is a common symptom of depression disorder. In rodents, liver-specific SAA1 overexpressing mice were considered a valuable model to study depression (Jang et al., 2017). The cytokine production of T helper 17 (Th17) cells was regulated by SAA (Lee et al., 2020), and the increase in Th17 production promoted by SAA may induce depressive-like behaviors in mice (Medina-Rodriguez et al., 2020). Thus, a segmented filamentous bacteria (SFB)/autoinducer-2 (AI-2)/SAA1-2/Th17 cell pathway that promoted depressive-like behavior was uncovered (Medina-Rodriguez et al., 2020). The evidence suggested that the SAA level may regulate depressive symptoms. Despite the lack of significant SAA level difference between the two groups, improvement of depressive symptoms in the second week was found associated with SAA level drop. As a matter of interest, this association was only present when rTMS treatment was combined. Consistently, another study in depression model mice found that rTMS reversed the down-regulation of astrocytes and inhibited high levels of IL-6, and IL-1β caused by chronic unpredictable mild stress (CUMS) in the hippocampus and prefrontal cortex (Zuo et al., 2022). Therefore, inhibition was also found on SAA in patients with the depressive disorder who were treated by rTMS combined with medicine, but not by medicine only. Inflammation was suggested to be associated with non-response to psychological therapy (Strawbridge et al., 2020), while it may be an indicator of rTMS therapy. Nevertheless, whether SAA is a state marker or a trait marker is still unclear due to the configuration results (Kling et al., 2007; Dahl et al., 2014). Thus, a follow-up study would be useful to answer this question.

It should be pointed out that no significant difference was found in testosterone levels between the two groups. Besides, no relationship was found between therapeutic effects and changes in testosterone levels either. Although lower testosterone level was associated with depression in men (McIntyre et al., 2006; Westley et al., 2015; Giltay et al., 2017), the results were inconsistent in women. A meta-analysis and Mendelian randomization study show that women with depression do indeed display significantly different serum levels of testosterone, which was most likely a manifestation of the disease itself (Maharjan et al., 2021). The meta-analyses indicate that testosterone appears to have a small antidepressant effect, while they do not provide strong support for the use of testosterone in depressive disorders in general (Dichtel et al., 2020; Dwyer et al., 2020). It is observed that most antidepressants can influence testosterone levels (Pavlidi et al., 2021), but the relationship between testosterone level and depressive symptom remission was not found in patients treated with the medicine. The hypothalamic-pituitary-gonadal (HPG) axis may offer a pathway to explain the impact of rTMS on this outcome (Crewther et al., 2022). Testosterone has been examined using rodent models of rTMS (Hedges et al., 2002, 2003). Nevertheless, these studies on rats failed to find an effect of HF–rTMS on testosterone levels. Therefore, testosterone might not be an indicative factor for rTMS or medicine treatments.

The limitations of this study are intrinsic to those of retrospective research conducted in a naturalistic setting. A sham rTMS group was not used to control for placebo effects. In addition, the present study used an atypical rTMS treatment protocol and did not control for concurrent medications or psychotherapy. The sample size of patients who completed baseline and second-week serological examinations was small, which limited our further analysis. At last, the follow-up time was only 2 weeks, which helped to understand the early onset but not the long-term effectiveness of rTMS therapy.

In conclusion, patients with depression benefit more from combined rTMS treatment with medicine in a naturalistic study. Changes in SAA but not testosterone level were related to depressive remission after 2 weeks of combined treatment. Future research is needed in the form of double-blind, randomized control trials that examines the relationship between SAA level and rTMS depression outcome.

## Data availability statement

The datasets generated and analyzed during the current study are not publicly available because permission is needed to access the database of the hospital, but they are available from the corresponding author on reasonable request. Requests to access these datasets should be directed to ZY, yuzhcoo@sina.com.

## Ethics statement

The studies involving human participants were reviewed and approved by Ethics Committee of Affiliated Mental Health Center and Hangzhou Seventh People’s Hospital, Zhejiang University School of Medicine. The patients/participants provided their written informed consent to participate in this study.

## Author contributions

All authors listed have made a substantial, direct, and intellectual contribution to the work, and approved it for publication.
